# Notes and Comments

**Published:** 1924-09

**Authors:** 


### NOTES AND COMMENTS.

### NEITHER DEAD NOR DYING.

"I WONDER if there is anyone who still thinks the Voluntary Hospital system is dead or dying," said the Prince of Wales at the recent meeting of the General Council of King Edward's Hospital Fund. He pointed to the significant figures of the situation. Even in 1920, when the crisis was at its worst, the London hospitals had an income of £2,400,000 as against £1,500,000 before the war. Since 1920 their income has increased to £2,850,000, without counting the £400,000 produced by the Combined Appeal. Their endowments have increased by nearly a million, and more than a million has been provided for extensions and improvements. Thus, over and above their annual income which has so greatly increased, the London hospitals have received in three years two millions and a half for special purposes, and this without counting additions during the same period to the investments of the King's Fund itself. Moreover, the beds have increased by 1,600, and will be much further increased when pending schemes of extension have been completed. This is a fine record, but records exist to be broken, and the work which lies before the hospitals of this country in the near future is enormous. It is work that will be faced with courage and determination, but it will not be accomplished rapidly.

### THE NEW HOUSING ACT.

MR. WHEATLEY'S Housing Act, which is designed to produce two millions and a-half of houses in the next fifteen years, is now on the Statute Book. If we may judge by the experience of the working of the Chamberlain Act, twelve months will suffice to indicate the extent to which it is likely to fulfil the roseate hopes of its author. That those hopes are, in the main, certain to be disappointed admits of no doubt whatever. The mesure is essentially unworkable, and is in the highest degree unlikely to produce better results than are already being obtained. Notwithstanding all the talk about agreements with the building trades, those industries are, in fact, pleadged to nothing. They will help on their own terms or not at all, and even the greater elasticity of apprenticeship of which they have talked is entirely inadequate. The Act even begins its career in the midst of a strike of building operatives, which may result in an increase in the cost of building, but in so far as the measure does operate it will load both the Treasury and the local authorities with a heavy weight of debt. The wisest thing Mr. Wheatley has done is to leave the Chamberlain Act to work freely for the remainder of its limited course.

### PRESERVING FOOD.

"WE have no hesitation in recommending that the addition of formaldehyde or any of its derivatives to food or drink should be absolutely and specifically prohibited." So says the interim report of the Food Preservatives Committee. So also said the Departmental Committee on Preservatives and Colouring Matters in Food as long ago as 1901; but still we have not "absolutely and specifically prohibited" the use as a food preservative of a substance which is "inimical to life and to vital processes of all kinds," is "a powerful protoplasmic poison," and "exerts an irritant action upon mucuous membranes." That is to say, it is about as mischievous a preservative as can be imagined. Almost every country that has legislated upon the purity of its food supply has banned the addition of formaldehyde to food. Argentina will not allow beef so treated even to be exported. How much longer is England going to lag behind? Dealers in food are almost as unanimous as public health authorities in condemning a practice which fell under censure nearly a quarter of a century ago. There is nothing to wait for and the sooner we make the application of formaldehyde to food a penal offence the better it will be for the health of the country.

### BROADCASTING HEALTH TALKS.

NOT to be outdone by the Americans, our own Ministry of Health is now broadcasting talks on health. The mind of the listener is not perhaps too receptive at ten o'clock at night, which was the hour at which Sir George Buchanan recently spoke on the subject of Epidemics and the work of the League of Nations. The topic was, however, handled in a simple and attractive manner by the speaker, who on such a subject is able to discourse with unrivalled authority. After a chat on the question of epidemics generally and of the ravages in particular of the mosquito and the rat-flea, Sir George Spoke of the enormous value of the work of the League of Nations in the pooling of medical experience and thought, whereby nations come to the relief of one another. He reminded his large audience of the way in which help has been rendered when "S.O.S." calls have gone up from Russia, Poland, and other areas. Epidemics are no respecters of boundaries between one country and another, and medical and sanitary science should know none. In this connection Sir George paid a tribute to the work of the Rockefeller Foundation, of whose beneficent activities we write elsewhere. The broadcasting of health talks is pregnant with great possibilities in the moulding of public opinion, and we congratulate the Ministry of Health and their Chief Medical Officer, Sir George Newman, who has himself broadcasted, on their promptitude in making use of modern scientific helps.

### THE WASHINGTON MATERNITY CONVENTION.

TO the deputation from the Standing Joint Committee of Industrial Women's Organisations which recently pressed upon him the importance of ratifying the Washington Maternity Convention, Mr. Wheatley had an opportunity of pointing out what we are already doing for the protection of mothers and their infants. Although the organisation has been in existence for a comparatively short time, there are already 2,200 infant welfare centres and 3,650 women employed on infant welfare work, in addition to 135 maternity hospitals and homes.

These are the means which, as we pointed out last month, have been mainly effective in reducing the infant death-rate from 150 to 69. Thus, as regards the children we have made excellent progress, but much remains to be done to reduce maternal mortality, which has remained practically stationary for a generation. We have learned how to save the children, but we are still sacrificing the mothers. This is the more deplorable since we are drying up the sources of infant life. To improve this unhappy state of things must be one of the chief and most immediate objects of welfare work. So long as employed women labour until the last practicable day before child-birth and return to work on the first possible day after it, maternal mortality will remain at a lamentably high level. Mr. Wheatley promised to bring the ratification of the Convention before the Government, but declined to accept the view of the deputation that the State ought to find the whole of the cost of carrying it out which would be a million and a half a year if it applied only to working mothers, and fifteen millions if it were applied all round. He was well justified in insisting that the local authorities should do their part. When one of the deputation rather unwisely asked the Minister of Health to believe that the Convention is being " effectively administered " in Russia, Poland and Austria, he drily replied that he had no such information. Nor has anyone else, so far, at all events, as Russia is concerned. So far as the world at large is aware, the only things that are " effectively administered " in Russia are the rope and the bullet.

### JERUSALEM AND GENEVA.

IN his preface to the fourth Report of the Joint Council of the Order of St. John of Jerusalem and the British Red Cross Society, Sir Arthur Stanley is able to record " an increasing indication in almost every part of the kingdom of revived interest in our work." The manifold activities of the Council continue to prosper and to expand—the number of patients, for instance, using ambulances was considerably larger last year than previously. Particularly valuable is the assistance given to those suffering from war disabilities. The total expenditure in this branch of work was £439,400 and the present monthly expenditure is over £10,000. That those helped are truly grateful is exemplified in a most practical manner. The men and widows who have been successful in their claims to pension, etc., refunded during last year the very substantial sum of £18,191. Nor are the ex-officers forgotten, over 500 of them being dealt with and an experimental House has been established in South-west London to accommodate advanced cases of tuberculosis Among them. Five million cigarettes and over 4,000 pounds of tobacco were issued to pensioner-patients ; concerts and amusements have been arranged and the men have had many little pleasures—gifts of games and gramophones—and the provision of bedside employment to while away weary hours. It is no surprise that so beneficial a work should be increasingly supported and that it should have attracted the services. of a devoted and tireless band of voluntary workers.

### IMPECUNIOUS STATE HOSPITALS.

BY a certain political school we have been assured, in accents of profound conviction, that impecuniosity and all the other disadvantages from which voluntary hospitals suffer, or are supposed to suffer, can only be removed by State control. Western Australia has a State Hospital Service, but, according to a report which has just been prepared by Mr. F. J. Huelin, the Secretary of the Department of Public Health, it is in a very unhappy position, chiefly because it is starved. Mr. Huelin's condemnation is sweeping indeed—" a lack of modern equipment, antiquated and unsuitable buildings, absence of central cultural surroundings, a low standard of ward equipment and furnishings, and an entire lack of hospital provision in many districts." Something approaching half the cost —£197,000—of running these sixty-one hospitals is provided by patients' fees, subscriptions, and donations. Thus there is a large amount of voluntary effort associated with the " State " hospitals of Western Australia. It would seem, indeed, that the State merely subscribes to certain of them, which are managed on much the same lines as our own voluntary hospitals, but there is no official control over them. The patients are expected to pay the hospitals as much as they reasonably can, but, for the most part, the medical staffs give their services gratuitously. Homes for incurables appear to be the only type of hospital which the State actually maintains. Why, then, call this a " State Hospital Service " ? It is very different from the ideal of the English Labour Party which—along with other people—would have something pointed to say about a hospital service which kept nurses on night duty for eighty hours a week, which is what happens in the smaller hospitals of Western Australia.

### THE SMOKE ABATEMENT BILL.

BEFORE Parliament rose the House of Lords passed through all its stages the Public Health (Smoke Abatement) Bill. It has not yet been before the Commons, and, looking at the congestion of business in " another place," it would be unwise to be sanguine about its chances. It is, however, a Government measure and therefore enjoys opportunities denied to legislation initiated by private members. As the Bill originally stood " black " smoke alone was incriminated, but Lord Newton succeeded in introducing an amendment, accepted by the Government, getting rid of that limitation. By a singular act of nepotism the Government sought to give itself permission to pour as much smoke as it pleased into the atmosphere from its own chimneys, but, so that the favouritism might not be too patent, it extended the privilege to public bodies and local authorities. Under pressure, however, it undertook, when the Bill reaches the Commons, to " endeavour to find words to meet the case." We hope that the Commons will take care that they do—it would be grotesque to grant a charter of pollution to public bodies while private persons were being prosecuted. One of Lord Newton's amendments will, however, need to be carefully watched. This amendment gives power to make local bye-laws requiring new private dwelling-houses to make such arrangements for warming and cooking as will prevent or reduce the emission of smoke. This might easily be an exceedingly oppressive provision, capable of being used for the prohibition of the healthy open fireplace and the substitution of less desirable methods of warming.

### "FOR REMEMBRANCE."

ROSEMARY, we know, is for remembrance. That is why Mr. F. de Burgh and Mr. Walter Stoneman have "collected and compiled" the handsome volume called "Rosemary," which has just been published at 7s. 6d. by Messrs. Sampson Low and Co. for the benefit of the "Not Forgotten" Association. That Society, as we hope all our readers know, exists to "anoint and cheer" the broken men, some recoverable and some irrecoverable who, six years after the end of the war, are still in hospital. There are 20,000 of them, and they all need comfort and visits, books, little presents and, most of them, occasional glimpses of the world outside. These are the works of mercy which the "Not Forgotten" Association performs by means of its members and friends. It has, moreover, its own hospital at Clapham, which is the centre of its beneficence. These treats and visits, cigarettes and other little alleviations of monotony cost money, and the more money we give the Association the better it can work, and the oftener it can cheer those who have suffered and still suffer—some of them, indeed, will suffer to the end of the chapter—for us and our safety. Hence this very attractive book, the profits of which will go to it. All manner of distinguished writers have contributed to it—Sir Sidney Low, who writes the Preface, Mr. G. K. Chesterton, Mr. John Galsworthy, Mr. John Drinkwater, Sir A. Conan Doyle, Mr. Arnold Bennett and Mr. Compton Mackenzie, and even now we have mentioned only a quarter of them. Their tales and poems and sketches make a stout and alluring volume, and every author has his portrait—if appearances go for anything, some of them have not recovered from their astonishment at finding themselves famous. We hope not only that the book will have a large sale, but that many of its readers will send little cheques to the Association at 86, Ladbroke Road, W. 11.

### THE LATEST PROFESSION.

DESPITE some recent improvements, the hospital libraries in this country are still, we fear, in a ludicrous condition of neglect, even when they are nominally in existence. The war helped to enforce the need of good books for patients, and the Red Cross library then founded did a good deal to supply the demand. None the less hospital libraries, whether for patients or nurses, are usually meagre and haphazard. It will, therefore, come as a surprise to many to learn that America, which makes every new departure into an elaborately organised business, has now established the profession of hospital librarian for women. One University, indeed, has started a five-year course of training for these new officials, beginning in September next! The first three years will be spent in collegiate work, the fourth at a training-school for librarians, the last at a special school where the "elements" of every subject pertaining to hospital and medical work, including "the therapeutic value of reading," will be studied. What any person who survives this course would think of our English hospital libraries is amusing to imagine, but we may take a hint from America to recognise at last that a hospital library should exist and not be shamed by comparison with any other hospital department

### THE FETISH OF FREE CHOICE OF DOCTOR.

AT the recent annual representative meeting of the British Medical Association, at Bradford, the subject of Free Choice of Doctor once more came up for discussion. The topic is one very dear to the doctors when they get on a public platform. It was moved that the principle of freedom of choice should be extended to Poor Law patients. On close examination it appeared to be out of the question to apply the principle to patients in infirmaries, and the resolution therefore took the form that free choice should, as far as possible, be recognised in the domiciliary attendance on Poor Law patients. In practice, with a very large percentage of the population, the operation of the principle of free choice means simply calling in the doctor round the corner, or the one who usually attends the person next door. It may be a little unkind to say so with such brutal frankness, but the fact cannot seriously be questioned. To apply the principle to the Poor Law patient with a cold in the head, while withholding it from his brother with serious illness in the infirmary, seems a little meaningless.

### TOO MUCH MEDICINE.

THE Minister of Health, in a communication to the Insurance Acts Committee of the British Medical Association, has drawn attention to the serious increase in the cost of medicines for insured persons, since the abolition in 1920 of the arrangement known as the "floating sixpence," under which the doctors had a pecuniary interest in keeping down the cost of drugs. The Minister reminds the Committee that it had been decided that before any action was taken in any part of the country of the kind contemplated in the Regulations, the Regional Staff of the Ministry should carry out a careful survey in order to obtain a comprehensive view of the facts in the country as a whole. An essential element of such a survey would be a series of interviews between officers of the Ministry and practitioners whose prescribing exhibits peculiarities that appear to merit investigation. The value of such interviews would be enhanced if no question could arise of disciplinary action on account of the prescribing under discussion, and the practitioner would not feel himself to be under the necessity of adopting a defensive attitude. The Minister gives this assurance, and he is justified in inviting the cordial co-operation of the profession in an endeavour to secure that the best results should ensue from such a survey. It is an unpleasant thought that so cynical a device as the "floating sixpence" might after all be necessary to secure that national economy in prescribing which is so essential, particularly when doctors, if one can get them to talk frankly, have so little faith in many of their compounds. It is for them to discourage the bottle-of-medicine habit, and to talk kindly but firmly to the well-meaning people who want to make a battle-cry of "Who seeks to rob the working-man of his drugs?"

### WARMING THE HOUSE.

RECENT excavations of Roman dwellings, and the opening up of their heating arrangements, have once more started a discussion upon the best method of keeping a house warm in winter—the season which so rarely leaves this island. The hot air of the hypocaust was carried beneath the floor, which was all very well when there was only one floor. The pavement was thus warmed, and we are asked to believe that this sufficed. But did it? That braziers were also used is certain, since fine examples of them have been found at Pompeii. Sir Gilbert Scott has adopted the Roman system for Liverpool Cathedral, and the advantages in the case of stone or marble floors are obvious. But English houses have more than one storey and the floors are usually of wood. Moreover, many people find it unpleasant to walk about, or rest their feet upon, warmed floors. That the open fire is dirty, wasteful and often inefficient we are all agreed, but the drawbacks of its substitutes are even more obvious and disagreeable. The open fire still holds the field as the most comfortable method of warming a room, and, by reason largely of the ventilation it creates, the healthiest. For these and other reasons it is the Briton's Ark of the Covenant upon which sacrilegious hands must not be laid. If we are content to have one half of a large room cold in the winter, why should not this national idiosyncracy be indulged? After all, the open fire never killed anybody. The radiator causes millions of colds every year, while a few hours of its baleful company sets even the nimblest brains a-simmering.

### HAY FEVER AND GLASS CASES.

HAY FEVER is so obstinate and distressing a complaint that it is almost inhuman to treat it lightly; but he would be a solemn person indeed, or a peculiarly severe sufferer, who failed to obtain some entertainment from a report on the subject which has just been issued by the United States Public Health Service. The document is as full of taboos as a work on tribal anthropology. If you are a hay-fever subject you must not live in a locality where there are many weeds—a neglected garden would, apparently, be fatal. During the fever season the sufferer should "avoid riding or driving into suburbs abounding in weeds"—walking into them would apparently be innocuous. He must keep away from electric fans, not go to the theatre or travel by railway, but if he must "take the cars" he should breathe through a moist handkerchief. Railway companies are requested kindly to keep their lines free from hay-fever weeds, which, we imagine, would be almost as easy as clearing the seashore of sand. Before choosing a district for residence there should be a consultation with a physician who knows all about hay fever. Mountains are good, but they must be not less than 6,000 feet up, which at once creates a sad difficulty for people condemned to live in flat countries. When the ideal home has been found, it must be surrounded by the right kind of trees. It is to be feared that these counsels of perfection for sufferers from hay fever will not avail them much. Most of them cannot live in the Alps or in glass cases or choose the trees that surround them, but must still look to medicine to relieve them, pending the discovery, which will happen sooner or later, of an effective preventive.

### MILK FROM TUBERCULOUS COWS.

IT is generally taken for granted that if tuberculosis in cattle could be eradicated there would be a decline in the incidence of this disease in man. The bovine type of tubercle bacillus having often been found in infants dying of tuberculosis, there is no doubt an element of truth in this assumption. On the other hand, there is a growing mass of evidence which proves that human tuberculosis is a matter of infection from one person to another, and a recent statistical survey of the incidence of bovine and human tuberculosis in Sweden by Dr. A. Lichtenstein brings out the importance of infection from man to man as compared with infection from cattle to man. Sweden is a largely agricultural country with a settled population which changes little from one generation to another; and while bovine tuberculosis is fairly common in the south and the midlands, it is practically unknown in the north. Dr. Lichtenstein found that the mortality from tuberculosis in children under the age of five was exceptionally high in the north, and when he classified the twenty-four counties in two equally large groups, according as the frequency of bovine tuberculosis was high or low, he found that in the twelve counties with a low frequency, the average incidence of bovine tuberculosis was 2.7 per 10,000 head of cattle. The corresponding figure for the twelve counties with a high frequency of bovine tuberculosis was 13.8 per 10,000. In the first group the death-rate from tuberculosis among children under the age of five was 12.7 per 10,000, while it was 14.8 per 10,000 in the second group. Thus, there was little difference in the incidence of fatal tuberculosis among children in the two groups of counties, although the incidence of bovine tuberculosis was about five times greater in the one than in the other. Dr. Lichtenstein also found that the infantile death-rate from tuberculosis in the various Swedish counties ran parallel with the incidence of fatal tuberculosis in human beings of all ages. The chief lesson of his investigations is not so much that bovine tuberculosis is a negligible factor in the development of the disease in man, as that the most serious source of infection is man himself.

